# Laser Pulpotomies’ Clinical and Radiographic Success in Primary Teeth by Type of Laser

**DOI:** 10.3390/children12111508

**Published:** 2025-11-06

**Authors:** Osama M. Felemban

**Affiliations:** Pediatric Dentistry Department, Faculty of Dentistry, King Abdulaziz University, Jeddah 21589, Saudi Arabia; omfelemban@kau.edu.sa

**Keywords:** laser pulpotomy, primary teeth, clinical success, radiographic outcomes, diode laser, low-level laser therapy, Nd:YAG, Er:YAG, Er,Cr:YSGG, CO_2_ laser

## Abstract

**Highlights:**

**What are the main findings?**

• Different types of lasers used in pulpotomy for primary teeth show varying levels of clinical success, with diode and Nd:YAG lasers demonstrating high success while evidence for CO_2_ lasers remains limited.
• Success rates and outcomes of laser pulpotomies may be influenced by laser type, device settings, and clinical protocols.

**What is the implications of the main findings?**

• Laser pulpotomy is potentially a viable alternative to traditional methods, but optimal results may depend on careful selection of laser type and clinical protocols.
• Further research is needed to establish clear guidelines and improve consistency in outcomes for laser pulpotomy in pediatric dentistry.

**Abstract:**

**Background/Objectives**: The aim of this literature review is to evaluate the clinical and radiographic success of various types of lasers used in pulpotomy procedures for primary teeth. **Methods**: A comprehensive literature search was conducted using PubMed and Web of Science databases, with no time limits applied. Keywords included various types of lasers (e.g., diode, Nd:YAG, Er:YAG, LLLT, CO_2_) and terms related to pulpotomy in primary teeth. The search was performed in August 2025. Studies were screened for eligibility based on inclusion and exclusion criteria, focusing on clinical trials and studies assessing long-term outcomes of laser pulpotomies. **Results**: The review included 36 studies, categorized by laser type. Diode lasers were the most commonly used laser and showed high clinical success rates (>85%) with various dressing materials. Nd:YAG lasers demonstrated positive long-term outcomes, while Er:YAG lasers had inconsistent results. Low-Level Laser Therapy (LLLT) and CO_2_ lasers also showed high clinical and radiographic success. The studies highlighted the variability in laser specifications and clinical protocols, making direct comparisons challenging. **Conclusions**: Laser pulpotomy offers a viable alternative to traditional methods, with diode and Nd:YAG lasers showing particularly high success rates. However, the lack of standardized protocols and the variability in study methodologies call for further research to establish clear guidelines for clinical practice.

## 1. Introduction

One of the vital pulp therapy techniques for primary teeth is pulpotomy, which aims to preserve the vitality of the radicular pulp when the coronal pulp is compromised by dental caries or trauma [1]. In pediatric dentistry, pulpotomy remains a commonly used therapeutic intervention for asymptomatic teeth with deep carious lesions resulting in pulp exposure [2]. Pulpotomy aims to preserve the primary tooth which will maintain chewing ability, arch length, appearance, and prevent speech issues, nutritional deficits, or malocclusion from early tooth loss [3]. The process of a typical pulpotomy involves the dentist removing the coronal portion of the pulp, followed by the application of a sterile cotton pellet to achieve hemostasis. A medication is then applied to the remaining radicular pulp tissue. The chamber is sealed to prevent leakage and microbial entry, and the tooth is typically restored with a stainless-steel crown as the treatment of choice, or other types of restorations such as composite, GIC, or Zirconia crowns [3,4].

The search for an ideal pulpotomy medicament has resulted in the implementation and ongoing evaluation of many agents. Worldwide, Formocresol (FC) has been established as the standard medicament for pulpotomy for over sixty years, primarily due to its ability to eradicate bacteria and fix tissues in addition to its ease of use, cost-effectiveness, and high clinical success rates [5,6]. However, concerns have been expressed about its potential to cause mutations, cancer, and immune system sensitization, particularly due to its formaldehyde content, resulting in a decrease in its popularity [7]. Over the years, several pulpotomy medicaments have been researched, with studies outlining their shortcomings. For example, Ferric Sulphate (FS) has uncertain long-term success, Calcium Hydroxide (CH) is prone to a compromised seal and high solubility, causing internal root resorption; Mineral Trioxide Aggregate (MTA) is costly and difficult to handle, and Zinc Oxide Eugenol (ZOE) has the potential to cause chronic inflammation when in contact with vital pulp tissue [3,5]. Agents such as Biodentine, glutaraldehyde, sodium hypochlorite (NaOCl), bioactive cements, and bone morphogenetic proteins have also been proposed and researched as pulpotomy medicament alternatives [8,9].

Laser pulpotomy represents a significant evolution in vital pulp therapy, leveraging light energy for tissue ablation, coagulation, sterilization, and biostimulation, rather than chemical fixation or pharmacological action [10,11]. During laser pulpotomies, laser ablation produces a superficial layer of coagulation necrosis that serves as a barrier to shield the deeper pulp from irritating materials, enabling the migration of fibroblasts for the formation of a dentinal bridge [12]. Laser energy rapidly coagulates blood proteins, facilitating efficient and predictable hemostasis of pulpal tissue, which is critical for successful medicament placement and reducing technique sensitivity [11,13]. Lasers exhibit potent bactericidal effects, decreasing the risk of subsequent infection, which may lower postoperative complications [14]. Moreover, laser-induced biostimulation stimulates cellular proliferation, differentiation, and growth factor synthesis, thus accelerating pulpal wound healing, facilitating reparative dentin formation, and potentially increasing long-term success [15,16].

Numerous systematic reviews and meta-analyses have reviewed laser pulpotomy therapy. However, to date, the vast majority have treated laser pulpotomy as a single intervention, combining studies of diode, Nd:YAG Er:YAG, Er,Cr:YSGG, low-level laser therapy (LLLT), CO_2_ and other laser types under a single category for analysis [17,18,19,20,21,22]. This methodological approach, albeit expedient for comparing “laser” versus “conventional” treatments, inadvertently masks the unique physicochemical properties, tissue interactions, and clinical efficacy profiles of each laser type. For example, diode lasers are highly effective in soft tissue surgery [11] and suitable for deep pulp disinfection and hemostasis [23]. Still, they may behave differently than Er:YAG lasers, which can remove hard dental tissues and are predominantly absorbed in water/hydroxyapatite [11]. Nd: YAG lasers provide deep coagulation and superior long-term outcomes in some studies, whereas LLLT and CO_2_ lasers, with distinct wavelengths and depth profiles, may differ significantly in clinical and radiographic success, as well as adverse event rates [24]. Since previous studies failed to distinguish between different laser types, the objective of this literature review is to evaluate the evidence for the clinical and radiographic success of pulpotomy procedures using each type of laser. The investigated laser types are diode, Nd:YAG, Er:YAG/Er,Cr:YSGG, CO_2_, and low-level laser devices. Evidence will be synthesized by laser type and clinical protocol to address gaps in the previous literature by highlighting the implications of laser selection on clinical outcomes and long-term prognosis.

## 2. Literature Search

A comprehensive and thorough literature search was conducted to ensure the inclusion of all relevant studies on laser pulpotomies in primary teeth. The search was carried out using PubMed and Web of Science databases. The database search was carried out in August 2025, and no time limits were applied to the search. Although other databases, such as Scopus and LILACS were unavailable, this restriction was lessened by examining and screening the references of the extracted studies, especially the systematic reviews that included the inaccessible databases. The following keywords were used for the literature search:

• Laser OR Erbium: Yttrium-Aluminum-Garnet (Er:YAG) OR Er:YAG OR Erbium, Chromium: Yttrium-Scandium-Gallium-Garnet (Er,Cr:YSGG) OR Er,Cr:YSGG OR Neodymium-Doped: Yttrium Aluminum Garnet (Nd:YAG) OR Nd:YAG OR Diode OR Low-level laser therapy OR LLLT OR CO_2_ laser.

and

• Pulp OR vital pulp treatment OR vital pulp therapy OR pulpotomy OR primary tooth OR primary teeth OR primary molar OR primary incisor OR primary dentition OR deciduous teeth OR deciduous dentition OR deciduous molar OR deciduous incisor.

**Figure 1:**
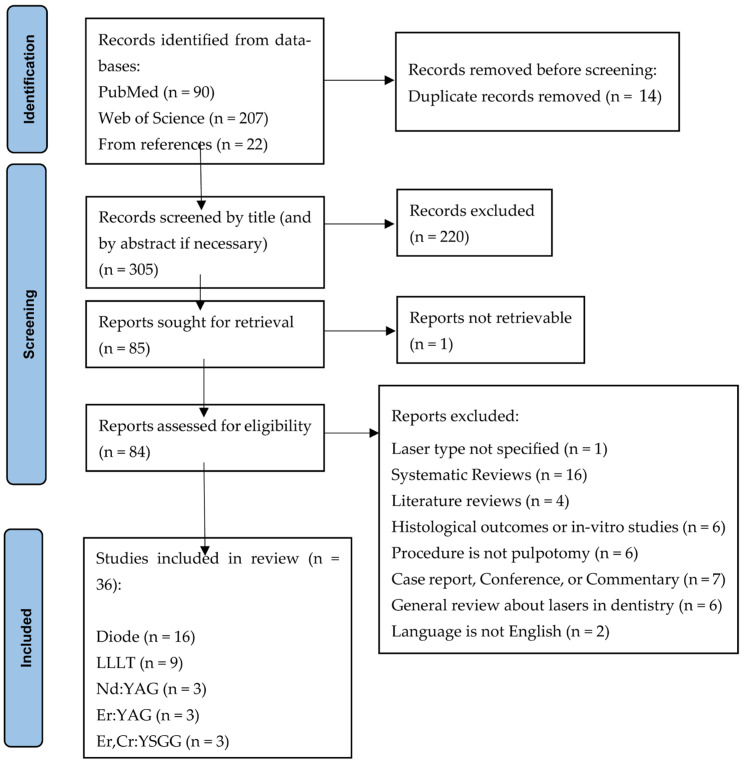
Selection of sources (flow diagram). LLLT Low level laser therapy; Nd:YAG Neodymium-Doped: Yttrium Aluminum Garnet; Er:YAG Erbium: Yttrium-Aluminum-Garnet; Er,Cr:YSGG Erbium, Chromium: Yttrium-Scandium-Gallium-Garnet.

Figure 1 shows a summary of the literature search process. The identified publications from the databases were imported into EndNote, and duplicates were removed. The articles were screened by title and, when necessary, by the abstract. After excluding irrelevant titles, a list of publications was compiled for full-text retrieval and subsequent eligibility assessment. The review’s inclusion criteria comprised clinical trials (randomized or non-randomized) and clinical studies (longitudinal studies) that assessed the long-term clinical and radiographic outcomes of laser pulpotomies in primary teeth, comparing them to other pulpotomy treatments and techniques. Systematic reviews and literature review articles were reviewed for their references, but they were not included in the final synthesis of the evidence. Exclusion criteria comprised animal studies, in vitro studies, case reports, and studies presenting only histological outcomes for laser pulpotomies. Excluded from the study were low-evidence study types, including interviews, conference abstracts, commentaries, editorials, letters, and opinions. Publications written in languages other than English were excluded. Thirty-six publications were included in the review. As this is not a systematic review, the included studies were not assessed for quality or risk of bias. The included studies were categorized by the type of laser used, and a single reviewer extracted the data and compiled it into summary tables for each type of laser. These tables summarized the year of publication, study site, number of subjects, age range, clinical criteria, pulp treatment, tooth restoration, duration of follow-up, and clinical and radiographic success rates.

## 3. Results

Almost all included studies applied the same initial clinical steps for the pulpotomy procedure. After applying a topical anesthetic, local anesthesia was administered. Teeth were then isolated using a rubber dam secured by an appropriate clamp. The pulpotomy procedure involved removing caries and opening the pulp chamber with a high-speed bur and water spray. The coronal pulp was amputated using either a sharp-spoon excavator or a slow-speed round carbide bur. The pulp chamber was then irrigated with distilled water, and bleeding was controlled by placing a wet or dry cotton pellet in the chamber for two to five minutes until hemostasis was achieved. The laser was subsequently applied, followed by placement of a capping material and the tooth was then restored.

### 3.1. Diode Laser

Table 1 compiles 16 studies examining the effectiveness of diode lasers in laser pulpotomies within diverse populations and clinical environments. The studies originated from multiple countries, such as Canada, Turkey, India, Iran, Taiwan, Malaysia, Iraq, and Croatia, and encompass subjects aged 2 to 10 years. Most of the studies were randomized clinical trials, except for two studies. Many studies had relatively small sample sizes. For instance, Saltzman et al., 2005, included only 16 subjects [25], and Ansari et al., 2018, had 14 subjects [26]. Other studies had relatively larger sample sizes, such as the study by Simunovic et al., which had 64 teeth per group [27], and Airpirala et al., which had 49–51 teeth per group [28]. The diode lasers used in these studies vary in wavelength, power, and mode of application. For instance, Saltzman et al., 2005, utilized a 980-nanometre (nm) diode laser, 3 watts (W), with a continuous pulse mode and multiple applications until hemostasis was achieved [25]. In contrast, Durmus and Tanbuga (2014) employed an 810 nm diode laser with a 50 megajoule (mJ), 1.5 W, 30 Hertz (Hz) setting and a 10 s exposure time [29].

Several studies compared diode lasers with other materials and techniques, such as FC, FS, and NaOCl. Comparative analysis often showed that diode lasers were either superior or comparable in terms of clinical and radiographic outcomes. The clinical success rates at the last follow-up varied across studies but were generally high. Studies that used ZOE, IRM (Intermediate restorative material), MTA, or Biodentine as the dressing material reported high clinical success rates (>85%) of diode laser pulpotomies. Radiographic success rates were also notable, with studies like Gupta et al., 2015, reporting a 100% success rate for the diode laser group [31]. However, the radiographic success rate was consistently lower than clinical success in most studies. The follow-up periods ranged from 1 to 24 months. Most of the studies limited their follow-up times to 12 months maximum. Only three studies followed up their subjects beyond 12 months and up to 24 months.

### 3.2. LLLT/Photobiomodulation

Eight studies in 9 publications reported on the use of LLLT in laser pulpotomies (Table 2). All the studies were RCTs, and five of them were in a split-mouth design. The studies had relatively small sample sizes, ranging from 15 to 43 teeth per group. Several wavelengths of LLLT were used, including 632 nm, 660 nm, 810 nm, and 820 nm. Some studies used the LLLT in contact mode, while others used it in non-contact mode. Different power and frequency settings and tips were used. Different exposure times were also used, ranging from 10 s to 4 min. Success rates were relatively high, ranging from 87% to 100% for clinical success and from 67% to 100% for radiographic success. When CH was used after LLLT as the dressing material, radiographic success was notably low. A key similarity between the two studies with the largest sample sizes (Alamoudi et al. [41] and Kaya et al. [42]) is that both studies included formocresol (FC) as a control group and assessed outcomes at 6 and 12 months, reporting high clinical success rates for both laser and FC groups. The study by Alamoudi et al., 2020 [41], specifically compared low-level laser therapy (LLLT) with formocresol, finding equivalent clinical success (96%) and slightly higher radiographic success for LLLT (100% vs. 98%) at 12 months, supporting LLLT as a biologically acceptable alternative [41]. In contrast, Kaya et al., 2022 [42] evaluated four techniques—calcium hydroxide with photobiomodulation (CH + PBMT), calcium hydroxide alone (CH), formocresol (FC), and mineral trioxide aggregate (MTA)—and found that FC and MTA had the highest success rates, while CH alone performed significantly worse. CH + PBMT showed comparable clinical success to FC and MTA but lower radiographic success, possibly due to the use of CH rather than the laser itself [42]. In summary, both studies support the effectiveness of laser-assisted pulpotomy but differ in their specific laser modalities and comparison groups, with Kaya et al. [42] providing a broader comparison and highlighting the limitations of calcium hydroxide alone.

### 3.3. Nd:YAG Laser

Three studies conducted in 2006 and 2007 utilized Nd:YAG in laser pulpotomies (Table 3). Two of the three studies compared Nd:YAG to FC, while the third study used Nd:YAG in all groups and then compared the clinical success between capping materials. All three studies appear to have used similar settings for Nd:YAG (1064 nm, 2 W, 20 Hz). However, two of the three studies employed the laser in non-contact mode. Furze and Furze observed clinical success rates of Nd:YAG laser pulpotomies between 90% and 100% depending on the capping material, though they did not clearly report radiographic outcomes [50]. Liu’s study provided both clinical (97%) and radiographic (94%) success rates for the Nd:YAG group, which were notably higher than those for the formocresol group [51]. Odabas et al. directly compared Nd:YAG laser with formocresol, finding similar clinical success (86% vs. 90%) but a lower radiographic success rate for the laser group (71% vs. 90%), with no statistically significant difference between the groups [52]. While all three support the clinical viability of Nd:YAG laser pulpotomy, Liu’s study stands out for demonstrating both high clinical and radiographic effectiveness, whereas Odabas et al. provide a more cautious interpretation due to the lower radiographic success observed.

### 3.4. Er:YAG Laser

Table 4 presents the two studies in three publications that investigated the Er:YAG laser pulpotomy. Both studies were RCTs and evaluated the effectiveness of Er:YAG laser-assisted pulpotomy in primary molars, focusing on long-term clinical and radiographic outcomes. The two studies appear to have used different settings for the laser and reported their settings differently. A key similarity is that both trials found Er:YAG laser pulpotomy to be effective, with clinical success rates above 85% at extended follow-up periods (36 months in Huth et al. [53], 24 months in Wang et al. [54]). However, the studies differ in their comparison groups and recommendations. Huth et al. compared Er:YAG laser with dilute formocresol, calcium hydroxide, and ferric sulphate, finding no significant differences in overall success between Er:YAG and formocresol, but noting that calcium hydroxide had a much higher failure rate [53]. In contrast, Wang et al. compared Er:YAG laser-assisted pulpotomy with MTA, reporting slightly higher—but not statistically significant—success rates for the laser group [54]. In summary, both studies support the use of Er:YAG laser in pulpotomy but differ in their comparative materials and clinical recommendations.

### 3.5. Er,Cr:YSGG Laser

Three recent RCTs investigating Er,Cr:YSGG laser pulpotomies were included (Table 5). All three studies implemented a randomized clinical trial design to investigate the effectiveness of Er,Cr:YSGG laser pulpotomy in primary molars, but they differ in their comparison groups and they used different laser settings. A common finding across the studies is that Er,Cr:YSGG laser pulpotomy achieved high clinical success rates and is comparable to conventional pulpotomy materials such as MTA, Biodentine, formocresol, and sodium hypochlorite. Ramanandvignesh et al. found no significant differences in success rates between Er,Cr:YSGG, MTA, and Biodentine at 9 months, suggesting similar efficacy among these approaches [56]. Fadhil and Noori extended this comparison, showing that both diode and Er,Cr:YSGG lasers performed as well as formocresol and sodium hypochlorite in short- and mid-term outcomes, supporting their use as biologically acceptable alternatives [39]. Sahin et al., while also reporting high clinical success for Er,Cr:YSGG, highlighted a notable difference in radiographic outcomes: the laser group had superior radiographic success compared to ferric sulphate and a herbal hemostatic agent, indicating a potential advantage of Er,Cr:YSGG in achieving long-term tissue health [57].

### 3.6. CO_2_ Laser

Only one study from 2002 was found to have studied CO_2_ laser pulpotomies. A prospective in vivo study conducted by Pescheck et al. in 2002 [58] assessed the long-term results of pulpotomy in 212 vital primary molars with deep caries that were treated under general anesthesia using a superpulsed CO_2_ laser, with a wavelength of 10.6 micrometre (µm), a power output of 3 W, and a 0.8-millimetre (mm) tip, for hemostasis. The laser treatment was followed by the application of ZOE, a Harvard cement base, and the placement of a stainless-steel crown. At 18-month follow-up, clinical success was 98% and radiographic success 92% [58].

## 4. Discussion

This review thoroughly examined and summarized published evidence on the clinical and radiographic success rate of laser pulpotomies using different laser types from 36 studies. The main findings indicate that laser pulpotomy is a promising alternative to traditional methods, with most laser modalities demonstrating high clinical success rates. Diode lasers were the most extensively studied and consistently showed clinical success rates above 85%, with radiographic outcomes also favourable, though slightly lower than clinical results. Nd:YAG laser pulpotomies exhibited excellent long-term clinical and radiographic success, often outperforming conventional medicaments such as formocresol. Er:YAG laser pulpotomies produced effective results, but their success rates were more variable, likely due to differences in clinical protocols and capping materials. Low-Level Laser Therapy (LLLT) and photobiomodulation also achieved high clinical and radiographic success, particularly when used with appropriate dressing materials, though outcomes were less favourable when paired with calcium hydroxide. Er,Cr:YSGG laser pulpotomies demonstrated comparable efficacy to conventional treatments, with some studies reporting superior radiographic outcomes. The single study on CO_2_ laser pulpotomy reported very high clinical (98%) and radiographic (92%) success.

At 18 months. Overall, laser pulpotomy protocols differ significantly, depending on device specifications, power, energy, and frequency. Still, the included studies show that the clinical and radiographic success rates for laser-assisted pulpotomy are often equal to or better than those achieved with conventional medicaments.

Clinical and radiographic success outcomes of laser pulpotomies vary by the type of laser used, comparison group, and capping agent. A recent meta-analysis and systematic reviews indicate that diode laser pulpotomy is as effective as, or possibly better than, FC or FS treatment [23]. Nd:YAG lasers have produced notably positive long-term outcomes (e.g., at 60 to 66 months), whereas Er:YAG performance appears more inconsistent when combined with various pulp capping agents [24]. Nevertheless, the distinction is rarely explicit in review conclusions, and recommendations for individualized laser selection are currently lacking. Limited research exists on how patient- or procedure-specific factors (such as age, tooth type, magnitude of pulpal bleeding, or operator experience) interact with laser modality to achieve the best results. The aggregation of all types of lasers in one category has two significant consequences: overlooking potential laser type-dependent differences in clinical outcomes, and the inability to establish proper treatment protocols for clinicians that take into account the unique features of each laser. This aggregation impedes the recognition of circumstances in which one laser modality might be preferred over others. Furthermore, there were inconsistencies between the studies in reporting laser specifications. Such variabilities make it challenging to standardize procedures and compare results across studies. Standardized protocols would help in better evaluating the efficacy of laser pulpotomies. The choice of dressing and restorative materials can influence the success of laser pulpotomies. Pulpal cells’ characteristics such as bioactivity, viability, and healing abilities are affected by the type of the pulpotomy medicament used [59]. Furthermore, it is advised that clinicians use materials that are effective in conjunction with the particular laser used so that it can be distinguished if any failure is the result of the laser type used or because of the capping material.

The primary rationale behind this literature review was to highlight that various types of lasers can produce differing effects on pulp tissue during pulpotomy procedures. Even when the same laser device is used, variations in settings and technical specifications can lead to distinct outcomes in pulp tissue response. Generally, the interaction between laser energy and pulp tissues is governed by several critical parameters that influence the accuracy, depth, and clinical results. For instance, lasers operating at different wavelengths can yield diverse effects on soft tissue. Wavelength, defined as the distance between successive peaks of the laser light wave, determines which tissue chromophores absorb energy. Shorter wavelengths penetrate deeper and primarily target pigmented proteins, whereas longer wavelengths are absorbed by water, resulting in more superficial tissue ablation [12,60]. Additionally, the emission mode, either continuous wave or pulsed, affects tissue outcomes. Pulsed emission allows for intermittent energy delivery and thermal relaxation, reducing collateral damage, while CW mode may cause excessive heat accumulation and deeper thermal effects [12,60]. The duration of laser exposure also plays a crucial role; shorter exposure times provide high peak power and precise ablation, whereas longer exposures increase the risk of thermal damage and tissue carbonization [12,60]. Furthermore, the choice between contact and non-contact modes influences energy delivery: contact mode offers tactile feedback and localized energy application, enhancing cutting efficiency, while non-contact mode disperses energy more broadly and is often preferred for coagulation or superficial treatments [12,60]. A thorough understanding and careful optimization of these parameters are essential for achieving safe, effective, and predictable outcomes in laser-assisted soft tissue procedures. Although standardizing these specifications in laser pulpotomies can be challenging, doing so may improve our understanding of the factors influencing the success rates of these procedures.

Pulpotomy is a vital tooth-saving method in pediatric dentistry. Although traditional treatments are effective, their drawbacks (mainly worries about toxicity, technique difficulty, or expense) have led to the growing use of laser-based treatments. Lasers offer clear mechanistic and clinical advantages, but neither current systematic reviews nor clinical guidelines adequately differentiate among the diverse types of lasers available. The safety profile of lasers is generally favourable when used according to manufacturer guidelines (e.g., with proper water cooling, minimized output power, and an appropriate distance from the target tissue) [61]. Serious adverse effects, such as excessive thermal damage or necrosis, are rare, contingent upon operator training and adherence to evidence-based protocols [62]. While laser pulpotomy has several benefits, it also has some drawbacks. For instance, it is expensive to initially purchase and to maintain the equipment, has a steep learning curve for optimal use, and lacks a universally accepted standard protocol [11]. These factors can impact accessibility and practicality, especially in private practice settings.

The primary limitation of the current review is that most of the included studies involve relatively small sample sizes, single-centre setups, short follow-up periods that rule out long-term assessments, and the possibility of operator or case-selection bias. Such drawbacks restrict the ability to generalize findings. Studies with larger sample sizes are necessary to validate the results and ensure they are representative of the broader population. A limitation of this review is that data extraction was conducted by a single reviewer. This approach may reduce methodological rigour and increase the potential for selection bias in the identification and interpretation of relevant studies. Moreover, the included studies utilized heterogeneous methodologies, laser parameters (power, frequency, mode), patient populations, outcome definitions, and follow-up durations, which confound direct comparisons and preclude laser-specific clinical recommendations. The reporting of adverse effects and complications was inconsistent across studies. Comprehensive reporting of any adverse effects is crucial to understanding the safety profile of lasers in pulpotomies. Addressing these limitations and gaps through larger, well-designed RCTs with standardized protocols and comprehensive reporting will enhance the understanding of the efficacy and safety of laser pulpotomies. This will ultimately contribute to establishing lasers as a reliable treatment modality in pediatric dentistry. Nevertheless, this literature review stands out for its comprehensive inclusion of diverse laser types and clinical protocols, rigorous literature search, and detailed comparison of long-term outcomes. By synthesizing evidence according to laser type, it provides practical insights for clinicians and highlights key gaps for future research.

## 5. Conclusions

Laser pulpotomies represent a significant advancement in vital pulp therapy for primary teeth, offering a viable alternative to traditional methods. Overall, the available limited evidence indicates that laser pulpotomies showed promise in improving the success rates and outcomes of pulpotomy procedures and demonstrated high clinical success rates and positive long-term outcomes. Diode and Nd:YAG lasers were reported to have high clinical success in pulpotomy, while Er:YAG and Er,Cr:YSGG lasers show more variable results. The evidence for CO_2_ lasers remains limited but promising. Low-Level Laser Therapy also demonstrates favourable outcomes, especially when paired with appropriate dressing materials. However, high-quality studies are needed to fully understand its potential and optimize its application in pediatric dentistry. Moreover, the variability in laser specifications, clinical protocols, and study methodologies made direct comparisons challenging. The lack of standardized protocols underscores the need for further research to establish clear guidelines for clinical practice.

## Figures and Tables

**Table 1:** Summary of clinical studies on Diode laser pulpotomies.

Author Year Location	n Subjects Age Study Design	n Teeth in Groups	Clinical Specifications and Procedures of Using the Diode Laser	Dressing Restoration	Follow Up in Months	Clinical Success at the Last Follow Up	Radiographic Success at the Last Follow Up
Saltzman et al., 2005 [25] Canada	16 subjects 3–8 years RCT Split-mouth	26 Diode + MTA 26 FC + ZOE	- 980 nm; 3 W; continuous pulse - 0.55 mm optical fibre - In contact with pulp tissue - Multiple applications until hemostasis achieved	MTA ZOE SSC	2, 5, 10, 16	13/13 (100%) Diode + MTA 7/7 (100%) FC + ZOE	11/13 (85%) Diode + MTA 5/7 (71%) FC + ZOE
Durmus and Tanbuga 2014 [29] Turkey	58 subjects 5–9 years RCT	40 Diode 40 FC 40 FS	- 810 nm; 50 mJ; 1.5 W; 30 Hz - 10 s exposure time - Fibre tip 1–2 mm away from tissue - Air cooling without water	ZOE + GIC SSC	1, 3, 6, 9, 12	40/40 (100%) Diode 39/40 (98%) FC 38/39 (93)% FS	30/40 (75%) Diode 35/40 (88%) FC 31/39 (80)% FS
Yadav et al., 2014 [30] India	37 subjects 4–7 years RCT	15 Diode 15 FS 15 ES	- wavelength NR; 3 W; Continuous wave - Non-contact mode for 2–3 s - 400 µm optical fibre - Multiple applications until hemostasis achieved	ZOE + GIC SSC	1, 3, 6, 9	15/15 (100%) Diode 13/15 (87%) FS 15/15 (100%) ES	12/15 (80%) Diode 12/15 (80%) FS 12/15 (80%) ES
Gupta et al., 2015 [31] India	30 subjects 4–10 years RCT	10 Diode 10 FS 10 ES	- 980 nm; 4 J/cm^2^; 3 W; Continuous pulse mode - 0.5 mm optical fibre - 2 min and 31 s exposure time - Multiple applications	ZOE SSC	3, 6, 9, 12	10/10 (100%) Diode 8/10 (80%) FS 8/10 (80%) ES	10/10 (100%) Diode 8/10 (80%) FS 8/10 (80%) ES
Niranjani et al., 2015 [32] India	60 subjects 5–9 years RCT	20 Diode 20 MTA 20 Biodentine	- 810 nm; 1.5 W - Pulse contact mode - 2 s application	ZOE SSC	3, 6	13/15 (87%) Diode 16/16 (100%) MTA 13/15 (87%) Biodentine	13/15 (87%) Diode 16/16 (100%) MTA 13/15 (87%) Biodentine
Joshi et al., 2017 [33] India	20 subjects 4–9 years RCT	20 Diode 20 FC	- 980 nm; 1.5 W; Continuous mode - 200 µm optical fibre - 2 s application - Contact mode	ZOE SSC	3, 6, 12	19/19 (100%) Diode 19/19 (100%) FC	15/19 (79%) Diode 11/19 (58%) FC
Ansari et al., 2018 [26] Iran	14 subjects 3–9 years RCT Split-mouth	20 Diode 20 FC	- 810 nm; 10 W; 20 Hz - 20 ms pulse duration - 40 ms interval time - Non-contact mode - 800 µm optical fibre - Multiple applications	ZOE SSC	6, 12	20/20 (100%) Diode 20/20 (100%) FC	18/20 (90%) Diode 20/20 (100%) FC
Kuo et al., 2018 [34] Taiwan	74 subjects 2–6.5 years Retrospective cohort	41 Diode 80 NaOCl 24 None	- 970 nm; 3 W; 5 Hz - Water cooling	ZOE SSC or composite	6, 24	32/32 (100%) Diode 56/63 (89%) NaOCl 16/16 (100%) None	30/33 (91%) Diode 44/67 (66%) NaOCl 14/16 (88%) None
Pratima et al., 2018 [35] Malaysia	40 subjects 4–9 years RCT	20 Diode +MTA 20 Diode +ZOE	- 980 nm; 2.5–3 W; continuous pulse - 0.5 mm optical fibre - In contact with pulp tissue - Multiple applications until hemostasis achieved		3, 6, 12	19/19 (100%) Diode + MTA 16/17 (94%) Diode + ZOE	19/19 (100%) Diode + MTA 16/17 (94%) Diode + ZOE
Shaikh et al., 2019 [36] India	NR subjects 4–8 years Design NR	20 Diode 20 Formocresol	NR	NR	1, 3, 6, 9	17/17 (100%) Diode 18/18 (100%) FC	16/17 (94%) Diode 16/18 (89%) FC
Pei et al., 2020 [37] Taiwan	70 subjects 2–8 years RCT	45 Diode 45 FC	- 915 nm; 2 W; 100 Hz - Contact mode for 1 s at each orifice 3 times until hemostasis	IRM SSC	3, 6, 9, 12	25/90 teeth after 12 m (93%) Diode (91%) FC	25/90 teeth after 12 m (79%) Diode (73%) FC
Aripirala et al., 2021 [28] India	98 subjects 4–8 years RCT	49 Diode 51 SGG	- 940 nm; 4 J/cm2; 2 W; 70–80 Hz - Gated pulse mode - 300 µm optical fibre	GIC SSC	3, 12	35/46 (76%) Diode 37/46 (80%) SG	24/46 (52%) Diode 30/46 (65%) SG
Satyarth et al., 2021 [38] India	40 subjects 6–8 years RCT	20 Diode + MTA 20 MTA	- 810 nm; 1.5 W; continuous mode - 2 s exposure - 200 µm optical fibre - In contact mode	MTA + GIC SSC	3, 6, 9	17/18 (94%) Diode + MTA 15/17 (88%) MTA	16/18 (89%) Diode + MTA 14/17 (82%) MTA
Simunovic et al., 2022 [27] Croatia	128 subjects 5–8 years RCT	64 Diode + Biodentine 64 Biodentine	- 980 nm; 3 W; continuous mode - 320 µm optical fibre - In contact mode for 2.5 min	Biodentine Composite or GIC	6, 12, 24	55/60 (92%) Diode + Biodentine 52/60 (87%) Biodentine	52/60 (87%) Diode + Biodentine 44/60 (73%) Biodentine
Fadhil and Noori 2024 [39] Iraq	34 subjects 4–8 years RCT	15 Diode 15 Er,Cr:YSGG 15 FC 15 NaOCl	- 940 nm; 2 W; continuous mode - 300 µm optical fibre - In contact mode for 1 s at each orifice	MTA SSC	6, 12	(100%) Diode (100%) Er,Cr:YSGG (96%) FC (88%) NaOCl	(95%) Diode (97%) Er,Cr:YSGG (85%) FC (88%) NaOCl
Simonska et al., 2025 [40] Croatia	37 subjects 4–8 years RCT	10 Diode +MTA 10 Diode + CH 10 MTA 10 CH	- 975 nm; 2 W; continuous mode - 10 s application until haemostasis	MTA + GIC CH + GIC Composite	1, 3, 6	7/9 (78%) Diode + MTA 7/10 (70%) Diode + CH 8/10 (80%) MTA 7/10 (70%) Ca(OH)_2_	

RCT Randomized clinical trial; MTA Mineral Trioxide Aggregate; FC Formocresol; FS Ferric sulphate; CH Calcium Hydroxide; ZOE zinc oxide eugenol; GIC glass ionomer cement; SSC stainless steel crown; ES Electrosurgery; NaOCl Sodium hypochlorite; NR not reported, IRM Intermediate restorative material; SG Simvastatin gel.

**Table 2:** Summary of clinical studies on LLLT/photobiomodulation pulpotomies.

Author Year Location	n Subjects Age Study Design	n Teeth in Groups	Clinical Specifications and Procedures of Using the LLLT/Photobiomodulation	Dressing Restoration	Follow Up in Months	Clinical Success at the Last Follow Up	Radiographic Success at the Last Follow Up
Golpayegani et al., 2010 [43] Iran	11 subjects 4–7 years RCT Split-mouth	23 LLLT 23 FC	- 632 nm; 4 J/cm^2^; continuous mode - 0.5 mm diameter optical fibre - 2 mm distance from pulp stums - 2 min 31 s exposure time	ZOE SSC	6, 12	15/15 (100%) LLLT 14/15 (93%) FC	10/15 (67%) LLLT 10/15 (67%) FC
Fernandes et al., 2015 [44] Brazil	n NR 5–9 years RCT	15 LLLT 15 LLLT + CH 15 CH 15 FC	- 660 nm; 2.5 J/cm^2^; 10 mW; 50–60 Hz - 320 mm diameter optical fibre; in contact mode - 0.04 cm^2^ focus beam - Irradiation time 10 s	ZOE RMGIC	6, 12, 18	15/15 (100%) LLLT 12/12 (100%) LLLT + CH 9/9 (100%) CH 15/15 (100%) FC	11/15 (73%) LLLT 9/12 (75%) LLLT + CH 6/9 (67%) CH 15/15 (100%) FC
Uloopi et al., 2016 [45] India	29 subjects 4–7 years RCT	20 LLLT 20 MTA	- 810 nm; 2 J/cm^2^; continuous mode - Applied over radicular stumps for 10 s	GIC SSC	3, 6, 12	16/20 (80%) LLLT 18/19 (95%) MTA	
Ansari et al., 2018 [46] Iran	40 subjects 3–9 years RCT split-mouth	40 LLLT + CEM 40 CEM 40 FC 40 FS	- 632 nm; 4 J/cm^2^ - 135 s exposure time	Zonalin SSC	6, 12	40/40 (100%) LLLT + CEM 39/40 (98%) CEM 40/40 (100%) FC 38/40 (95%) FS	40/40 (100%)LLLT + CEM 38/40 (95%) CEM 40/40 (100%) FC 37/40 (93%) FS
Alamoudi et al., 2020 [41] Nadhreen et al., 2021 [47] Saudi Arabia	36 subjects 5–8 years RCT split-mouth	53 LLLT 53 FC	- 810 nm; 4 J; 6.7 J/cm^2^; 3 W; 5 W/cm^2^ continuous pulse mode - 200 µm diameter optical fibre - 2 mm away from pulp tissue - 105 µm focus beam - 40 s irradiation time	IRM SSC	6, 12 3, 9	49/51 (96%) LLLT 49/51 (96%) FC	51/51 (100%) LLLT 50/51 (98%) FC
Yavagal et al., 2021 [48] India	4–7 years RCT Split-mouth	34 PBM 34 FC	- 660 nm; 36 mW - Non-contact mode - 4 min exposure time	GIC SSC	9	32/34 (94%) PBM 33/34 (97%) FC	32/34 (94%) PBM 20/34 (59%) FC
Kaya et al., 2022 [42] Turkey	94 subjects 5–8 years RCT	43 PBM + CH 43 CH 43 FC 43 MTA	- 820 nm; 2.5 J/cm^2^; 10 mW - 12 s exposure time - 1 mm from target tissue - 0.047 cm^2^ spot size area	Zinc phosphate cement except FC covered with ZOE	6, 12	(87%) PBM + CH (71%) CH (97%) FC (97%) MTA	(73%) PBM + CH (45%) CH (92%) FC (95%) MTA
Haghgoo et al., 2023 [49] Iran	34 subjects 3–8 years RCT split-mouth	34 PBM + MTA 34 PBM + CH 34 PBM + CEM	- 632 nm; 4 J/cm^2^; 30 mW; 1–50 Hz - Non-contact (2 mm distance); - Photobiomodulation mode - Continuous-wave mode with 0.5 cm^2^ - Cross-sectioned area of the nozzle tip - Laser was irradiated for 75 s	ZOE SSC	6, 12, 18, 36	29/30 (97%) PBM + MTA 25/30 (83%) PBM + CH 29/30 (97%) PBM + CEM	28/30 (93%) PBM +MTA 20/30 (67%) PBM + CH 28/30 (93%) PBM + CEM

LLLT Low level laser therapy; PBM Photobiomodulation; RCT Randomized clinical trial; MTA Mineral Trioxide Aggregate; FC Formocresol; FS Ferric sulphate; CH Calcium Hydroxide; CEM Calcium Enriched Mixture; ZOE zinc oxide eugenol; GIC glass ionomer cement; SSC stainless steel crown; NR not reported, IRM Intermediate restorative material; Zonalin (resin-modified zinc oxide eugenol).

**Table 3:** Summary of clinical studies on Nd:YAG laser pulpotomies.

Author Year Location	n Subjects Age Study Design	n Teeth in Groups	Clinical Specifications and Procedures of Using the Nd:YAG Laser	Dressing Restoration	Follow Up in Months	Clinical Success at the Last Follow Up	Radiographic Success at the Last Follow Up
Furze and Furze. 2006 [50] Argentina	NR Prospective clinical study	35 Nd:YAG + CH 20 Nd:YAG + CH + Iodoform 10 Nd:YAG + GIC	- 1064 nm; 2 W; 20 Hz - 10 s application - 2–3 mm distance from the stumps	CH CH + Iodoform GIC NR	12	33/35 (94%) Nd:YAG + CH 20/20 (100%) Nd:YAG + CH + Iodoform 9/10 (90%) Nd:YAG + GIC	NR
Liu. 2006 [51] Taiwan	55 subjects 4–7 years Design NR	68 Nd:YAG 69 FC	- wavelength NR; 100 mJ; 2 W; 20 Hz - 320 µm optical fibre	IRM SSC or composite	Nd:YAG 6–64 m FC 9–66 m	66/68 (97%) Nd:YAG 59/69 (86%) FC	64/68 (94%) Nd:YAG 54/69 (78%) FC
Odabas et al., 2007 [52] Turkey	30 subjects 6–9 years Design NR	21 Nd:YAG 21 FC	- 1064 nm; 100 mJ; 2 W; 20 Hz - Non-contact	IRM SSC or amalgam	1, 3, 6, 9, 12	18/21 (86%) Nd:YAG 19/21 (90%) FC	15/21 (71%) Nd:YAG 19–21 (90%) FC

FC Formocresol; CH Calcium Hydroxide; GIC glass ionomer cement; SSC stainless steel crown; NR not reported, IRM Intermediate restorative material.

**Table 4:** Summary of clinical studies on Er:YAG laser pulpotomies.

Author Year Location	n Subjects Age Study Design	n Teeth in Groups	Clinical Specifications and Procedures of Using the Er:YAG Laser	Dressing Restoration	Follow Up in Months	Clinical Success at the Last Follow Up	Radiographic Success at the Last Follow Up
Huth et al., 2005 [55] Huth et al., 2012 [53] Germany	107 subjects ≤ 8 years RCT	47 Er:YAG 50 FC 44 CH 50 FS	- 2490 nm; 180 mJ/pulse; 2 Hz - Pulsating mode - Without wate cooling - Mean number of laser pulses per tooth was 31.5 ± 5.9, equally distributed to each pulp stump	IRM SSC or composite	6, 12, 18, 24, 36	(89%) Er:YAG (92%) FC (75%) CH (97%) FS	(73%) Er:YAG (72%) FC (46%) CH (76%) FS
Wang et al., 2022 [54] China	40 subjects 3–6 years RCT	50 Er:YAG + MTA 50 MTA	- 2940 nm; 20 mJ/pulse; 0.64 J/cm^2^; 9.6 W/cm^2^; 15 HZ - Pulse duration of 300 ms - Spot with a diameter of 1 mm - 1 mm distance from pulp tissue	MTA + GIC SSC	6, 12, 18, 24	43/48 (90%) Er:YAG + MTA 39/47 (83%) MTA	

RCT Randomized clinical trial; MTA Mineral Trioxide Aggregate; FC Formocresol; FS Ferric sulphate; CH Calcium Hydroxide; GIC glass ionomer cement; SSC stainless steel crown; IRM Intermediate restorative material.

**Table 5:** Summary of clinical studies on Er,Cr:YSGG laser pulpotomies.

Author Year Location	n Subjects Age Study Design	n Teeth in Groups	Clinical Specifications and Procedures of Using the Er,Cr:YSGG Laser	Dressing Restoration	Follow Up in Months	Clinical Success at the Last Follow Up	Radiographic Success at the Last Follow Up
Ramanandvignesh et al., 2020 [56] India	45 subjects 4–9 years RCT	18 Er,Cr:YSGG 18 MTA 18 Biodentine	- Wavelength NR; 75–100 mJ or 100–120 mJ; 1–1.5 W or 1–1.8 W; 10–15 Hz - Water spray - 600 µm tip diameter - 60 s - Repeated three to four times	ZOE SSC	3, 6, 9	13/16 (81%) Er,Cr:YSGG 14/17 (82%) MTA 17/17(100%) Biodentine	13/16 (81%) Er,Cr:YSGG 14/17 (82%) MTA 17/17(100%) Biodentine
Fadhil and Noori 2024 [39] Iraq	34 subjects 4–8 years RCT	15 Er,Cr:YSGG 15 Diode 15 FC 15 NaOCl	-2780 nm; 1.5 W; 50 Hz; soft tissue -20% air cooling; no water -tip-type MZ6 was applied for 10 s until a fixed char layer formed over the canal orifice	MTA SSC	6, 12	100% Er,Cr:YSGG 100% Diode 96% FC 88% NaOCl	97% Er,Cr:YSGG 95% Diode 85% FC 88% NaOCl
Sahin et al., 2025 [57] Turkey	65 subjects 5–9 years RCT	27 Er,Cr:YSGG 27 FS 27 HHA	-Wavelength NR; 25 mJ; 0.5 W; 50 Hz -Non-caontact 3–4 mm away from tissue -10 s application time -600 mm tip diameter (MZ6)	ZOE SSC	3, 6, 9, 12	26/26(100%) Er,Cr:YSGG 24/25 (96%) FS 26/26 (100%) HHA	25/26 (96%) Er,Cr:YSGG 19/25 (76%) FS 19/26 (73%) HHA

RCT Randomized clinical trial; FC Formocresol; FS Ferric sulphate; HHA Herbal haemostatic agent; ZOE zinc oxide eugenol; SSC stainless stell crown; NaOCl Sodium hypochlorite.

## Data Availability

No new data were created or analyzed in this study.

## Abbreviations

The following abbreviations are used in this manuscript:

Er:YAG	Erbium: Yttrium-Aluminum-Garnet
Er,Cr:YSGG	Erbium, Chromium: Yttrium-Scandium-Gallium-Garnet
Nd:YAG	Neodymium-Doped: Yttrium Aluminum Garnet
LLLT	Low-level laser therapy
RCT	Randomized clinical trial
MTA	Mineral Trioxide Aggregate
FC	Formocresol
FS	Ferric sulphate
CH	Calcium Hydroxide
ZOE	zinc oxide eugenol
GIC	glass ionomer cement
SSC	stainless steel crown
ES	Electrosurgery
NaOCl	Sodium hypochlorite
IRM	Intermediate restorative material
CEM	Calcium Enriched Mixture
HHA	Herbal haemostatic agent
SG	Simvastatin gel
NR	not reported
